# Comparison of spinal curvature parameters as determined by the ZEBRIS spine examination method and the Cobb method in children with scoliosis

**DOI:** 10.1371/journal.pone.0200245

**Published:** 2018-07-09

**Authors:** Mária Takács, Zsanett Orlovits, Bence Jáger, Rita M. Kiss

**Affiliations:** 1 Department of Orthopedics, MÁV Hospital Szolnok, Szolnok, Hungary; 2 Institute of Mathematics, Budapest University of Technology and Economics, Budapest, Hungary; 3 Department of Structural Engineering, Budapest University of Technology and Economics, Budapest, Hungary; 4 Department of Mechatronics, Optics and Mechanical Engineering Informatics, Budapest University of Technology and Economics, Budapest, Hungary; Medical College of Wisconsin, UNITED STATES

## Abstract

**Background and purpose:**

The most common and gold standard method to diagnose and follow-up on scoliosis treatment is to capture biplanar X-ray images and then use these to determine the sagittal frontal spinal curvature angles by the Cobb method. Reducing exposure to radiation is an important aspect for consideration, especially regarding children. The ZEBRIS spinal examination method is an external, non-invasive measurement method that uses an ultrasound-based motion analysis system. The aim of this study is to compare angle values of patients with adolescent idiopathic scoliosis (AIS) determined by the ZEBRIS spine examination method with the angle values defined by the gold standard Cobb method on biplanar X-ray images.

**Methods:**

Subjects included 19 children with AIS (mean age 14.5±2.1 years, range 8–16 years, frontal plane thoracic Cobb angle 19.95±10.23°, thoracolumbar/lumbar angle 16.57±10.23°). The thoracic kyphosis and lumbar lordosis in the sagittal plane and the thoracic and lumbar scoliosis values were calculated by the Cobb method on biplanar X-ray images. The sagittal frontal spinal curvature angles were calculated from the position of the processus spinosus of 19 vertebrae, as determined by the ZEBRIS spine examination method. The validity of the ZEBRIS spine examination method was evaluated with Bland-Altman analyses between the sagittal and frontal spinal curvature parameters calculated from data determined by the ZEBRIS spine examination method and data obtained by the Cobb method on the X-ray images.

**Results and discussion:**

Thoracic spinal curvature angles in sagittal and in frontal planes can be measured with sufficient accuracy. The slopes of the linear regression lines for thoracic kyphosis (*TK*) and thoracic scoliosis (*TSC*) are close to one (1.00 and 0.79 respectively), and the intercept values are below 5 degrees. The correlation between the *TK* and *TSC* values determined by the two methods is significant *(p =* 0.000) and excellent (*r*_*TK*_ = 0.95, *r*_*TSC*_ = 0.85). The differences are in the limit of agreement. The lumbar lordosis (*LL*) in the sagittal plane shows a very good correlation (r_*LL*_ = 0.76); however the differences between the angles determined by the two methods are out of the limit of agreement in patients with major lumbar lordosis (*LL*≥50°). The thoracolumbar/lumbar spinal curvature angles in the frontal plane determined by ZEBRIS spine examination were underestimated at curvatures larger than 15°, mainly due to the rotational and pathological deformities of the scoliotic vertebrae. However, the correlation between lumbar scoliosis (*LSC*) values determined by the two methods is significant (*p* = 0.000) and excellent (*r*_*LSC*_ = 0.84), the slopes are below one (0.71), the intercept values are below 5 degrees, and the differences between the angles determined by the two methods are within the limits of agreement. We could conclude that ZEBRIS spinal examination is a valid and reliable method for determination of sagittal and frontal curvatures during the treatment of patients with scoliosis. However, it cannot replace the biplanar X-ray examination for the visualization of spinal curvatures in the sagittal and frontal planes and the rotation of vertebral bodies during the diagnosis and annual evaluation of the progression.

## Introduction

There are several spinal deformities that can develop in childhood, of which, scoliosis is the most common and the most serious. Of 10-16-year-old children, 2 to 4% are suffering from adolescent idiopathic scoliosis (AIS) [1]. This new type of classification emphasizes the fact that scoliosis is a complex, three-dimensional deformity and sagittal spinal curvatures play an important role in the stability of the spine [2,3]. The basic components of deformity are thoracic kyphosis (*TK*) and lumbar lordosis (*LL*) (sagittal plane), inclinations (frontal and sagittal planes) and axial rotation (transverse plane), i.e., the vertebral bodies rotate towards the convex side and the spinous processes towards the concave side [4,5]. AIS patients were shown to have a distinct asymmetrical intravertebral deformity, with the maximum being in the apical region of the curve [6].

Biplanar X-ray images of the full spine are recommended for the visualization of spinal curvatures in the sagittal and frontal planes and the rotation of vertebral bodies, initially, when the diagnosis is made and then annually to evaluate the progression of the spinal deformities [7,8]. The Cobb method is the most widespread method used for determining the degree of scoliosis in the frontal plane, and the degree of thoracic kyphosis and lumbar lordosis in the sagittal plane [9,10].

To reduce radiation exposure, the use of low-radiation devices has become more common in determining spinal curvatures, such as EOS 2D/3D devices, which are suitable for determining the Cobb angles [11–17]. Follow-up should be regular and frequent for children with scoliosis, which significantly increases their level of radiation exposure. Higher levels of exposure increase the risks of lung cancer, breast cancer and leukaemia in patients with AIS [18,19]. The recommendation by the Society on Scoliosis Orthopedic and Rehabilitation Treatment (SOSORT) in 2014 proposes the use of alternative, non-radiation devices [20]. The flexiruler, the goniometer, the kyphometer, ultrasound-based systems such as the Scoliscan, rasterstereographic systems and the Spinal Mouse (SM) are the best-known non-invasive devices to determine spinal curvatures through the skin [21,22, 23–30, 31–36].

Aroeira et al [37] used computerized photogrammetry with a digital camera to establish the frontal spinal curvature angles. Aroeira et al established via the measurement of 16 young adults with AIS that the average difference between the two methods is 2.9° in the thoracic region and 5.1° in the lumbar region between the frontal angles determined by the gold standard Cobb method on frontal X-ray images and the angles as determined by computerized photogrammetry. The differences are not significant. The test- retest reliability and the accuracy determined by Bland-Altman method are unknown [37].

Schmid et al [38] established via the measurement of 10 children with AIS that skin marker based motion capture techniques can be used for the non-invasive assessment of spinal curvature angles in the sagittal and frontal planes in patients with AIS. However, the accuracy of measurement is influenced by the rotational deformities of the scoliotic vertebrae, and the angles in the frontal plane were systematically underestimated. Schmid et al. [38] found that using radio-opaque markers during radiographic measurements and the thickness of the soft tissue could significantly influence the accuracy of the determination of spinal curvatures.

The previous studies established that the frontal plane spinal curvatures derived from the processus spinosus significantly underestimated the Cobb angle [39], and the malrotation of vertebral bodies might lead to an underestimation of the frontal plane spinal curvature [38,40,41]. However, the motion analysis system is recommended for a comprehensive, non-invasive evaluation of treatment effects [38,40,41].

The ZEBRIS ultrasound-based motion analysis system (ZEBRIS Medizintechnik GmbH, Isny, Germany) is an external non-invasive ultrasonography-based system, which determined the spatial coordinates of the spinosus processus in the local coordinates system and defined three system receivers by triangulation (hereinafter referred to as ZEBRIS spine examination method) [42–46]. The test-retest reliability of the ZEBRIS spine examination method were determined by two independent, experienced examiners in the cases of 23 children with AIS; initial measurements, and those repeated three weeks later, were performed by both examiners. The test-retest reliability of thoracic kyphosis is excellent, with an intraobserver reliability of 0.958, and interobserver reliability of 0.948. The test-retest reliability of lumbar lordosis is very good, with intraobserver reliability of 0.814, interobserver reliability of 0.793 [47].

To our knowledge, regarding patients with AIS, the angle values determined by the ZEBRIS spine examination method have never been compared with the angle values defined by the gold standard Cobb method on biplanar X-ray images. The aim of the present study is to evaluate the static validity of the ZEBRIS spine examination method in the determination of spinal curvatures in the sagittal and frontal planes in patients with AIS using biplanar radiography. In this study, the effect of soft tissue thickness in the lumbar region could not be analysed by radiological methods. Schmid et al. [38] analysed the effect of soft tissue thickness using a radio-opaque marker. We did not have the opportunity to carry out such examinations in our study, so we assumed that the effect of soft tissue thickness could be modelled using the BMI. The effect of soft tissue thickness could be modelled by the Pearson correlation coefficient between the body mass index (BMI) specifically determined for children [48,49] and the difference between the angular values determined by the two methods. Our hypothesis is the following: the sagittal plane spinal curvatures determined by the Cobb method in biplanar radiological measurements, and those defined by the ZEBRIS spine examination method do not differ significantly; the frontal plane spinal curvatures are underestimated; however, the correlation between these values is at least good.

## Materials and methods

### Ethics statement

The research was approved by the Research Ethics Committee of MÁV Hospital in Szolnok (number: FI/5-93/2007). Every participant and their parents had received detailed oral and written information before they signed their informed consent.

### Subjects

Thirty-seven patients at MÁV Hospital with diagnosed AIS were scheduled for a routine orthopaedic examination by radiography in September 2016 in the Orthopaedic Department. Inclusion criteria for participation in the present study were the diagnosis of AIS (types 1 and 3 according to Lenke’s classification) [6], and age between 8–16 years. Exclusion criteria were inequalities or congenital abnormalities (spina bifida, hemivertebra, etc.), spinal curvature deformities other than those due to idiopathic scoliosis, and previous surgical interventions on the spine. According to the inclusion and exclusion criteria, 19 patients with AIS (17 females, 2 males) were included from the initial pool of 37 patients (Table 1). Mean age was 14.5±2.1 years (range: 8–16 years), mean body mass was 50.5±10.6 kg (range: 30–67 kg) and mean height was 165.4±11.1 cm (range: 140–182 cm). BMI was calculated using two methods: the conventional method (body mass divided by height squared) (18.27±2.37 kg/m^2^, range 14.60–23.70 kg/m^2^), and according to the recommendations of Ogden [48,49] (34.68±20.71%, range: 5–80%) (Table 1).

**Table 1:** Subject demographics data.

ID	gender	age	body height	body mass	Body Mass Index (BMI)		Lenke type [6]
		[years]	[cm]	[kg]	[kg/m^2^]	BMI percentile [%] [48,49]	
1	female	15	163	53	19.90	51	3
2	female	15	178	58	18.30	28	1
3	female	16	175	63	20.60	54	3
4	female	13	164	48	17.80	35	1
5	female	16	166	55	20.00	46	3
6	female	16	176	60	19.40	37	3
7	male	13	177	55	17.60	33	1
8	male	11	140	30	15.30	14	1
9	female	16	159	44	17.40	14	3
10	female	14	148	32	14.60	32	1
11	female	14	162	47	18.30	34	1
12	female	15	173	60	20.00	52	1
13	female	14	165	54	19.80	55	1
14	female	16	170	48	16.60	6	1
15	female	16	182	54	16.30	5	3
16	female	16	162	42	16.00	5	1
17	female	16	168	67	23.70	80	3
18	female	16	167	58	20.80	57	1
19	female	8	147	32	14.80	21	1

### Radiological measurements

**Fig 1:**
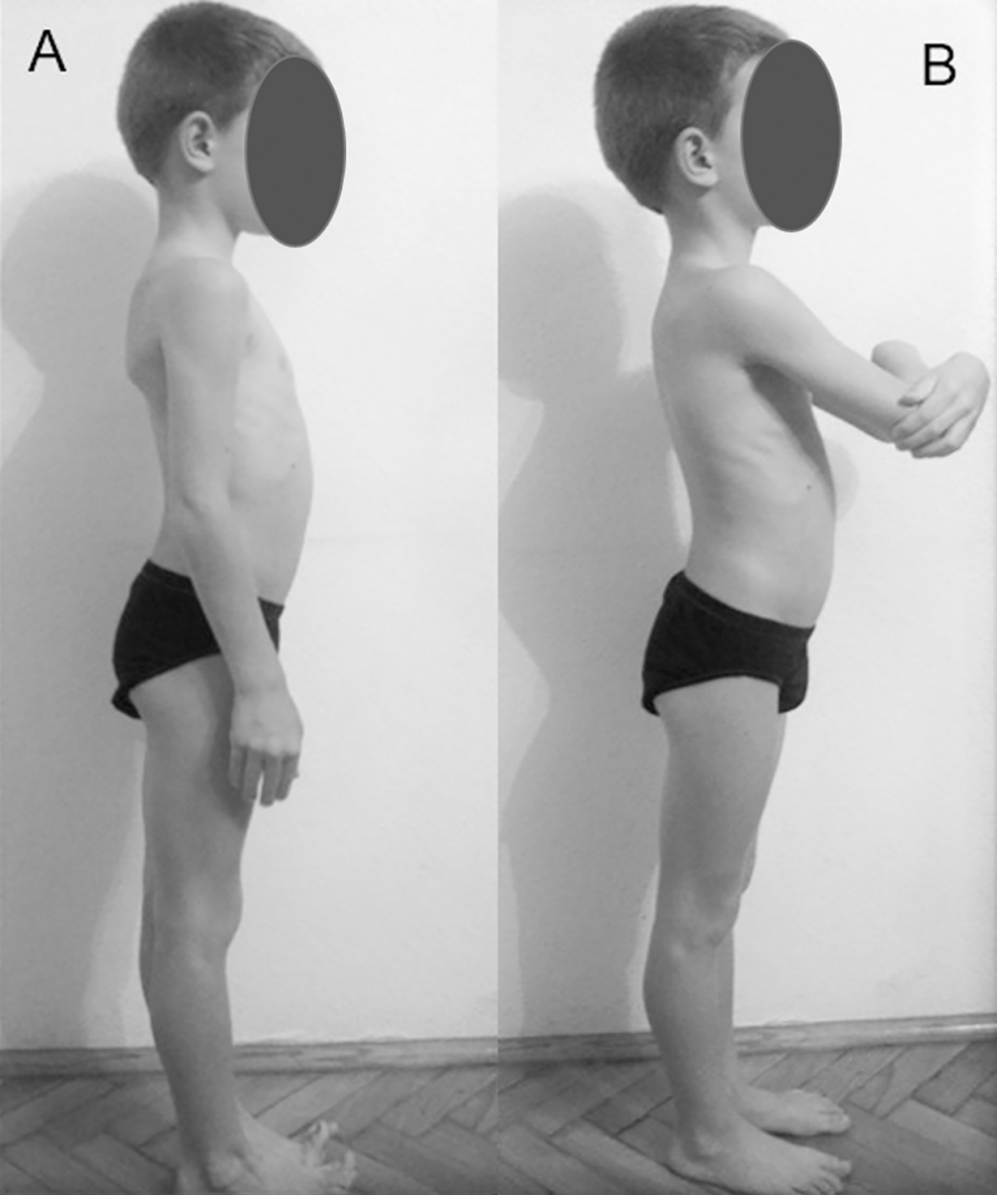
Standing positions. A: standing position with lowered arms: a natural standing position with the arms lowered loosely at the sides of the trunk. B: standing position with raised arms: a natural standing position with the arms raised to 45° in the sagittal plane and with hands grabbing the opposite side elbows.

Standard biplanar (posterior-anterior and lateral) radiographic examinations of the full spine were taken by a digital X-ray (Siemens Luminous Fusion Digital X-ray 2015/31030):

- Posterior-anterior (PA) X-ray beams were used for frontal full spine X-ray images [20]. Children were asked to stay in a natural straight standing position and to keep their arms loosely at the sides of the trunk (Fig 1A) (Hereinafter referred to as standing position with lowered arms).

- From left to right, lateral X-ray beams were used for sagittal full spine X-ray images. Previous studies [50–55] have proved that the evaluation of the images is the most difficult and most inaccurate when the arms are positioned at the sides of the trunk. The results improve when the arms are folded and raised to 45° [52,53]. Accordingly, the patients were asked to stay in a natural standing position, to stretch their knees and hips, to raise their arms to 45° and to grab the opposite side elbows (Fig 1B) (Hereinafter referred to as standing position with raised arms).

### ZEBRIS WinSpine measurement

The ZEBRIS spine examination was performed directly after the radiological and orthopaedic examinations. In the case of the ZEBRIS spine examination method the spatial positions of the spinous processes were determined by the ultrasonography-based ZEBRIS CMS-HS motion analysis system (ZEBRIS Medizintechnik GmbH, Isny, Germany)

**Fig 2:**
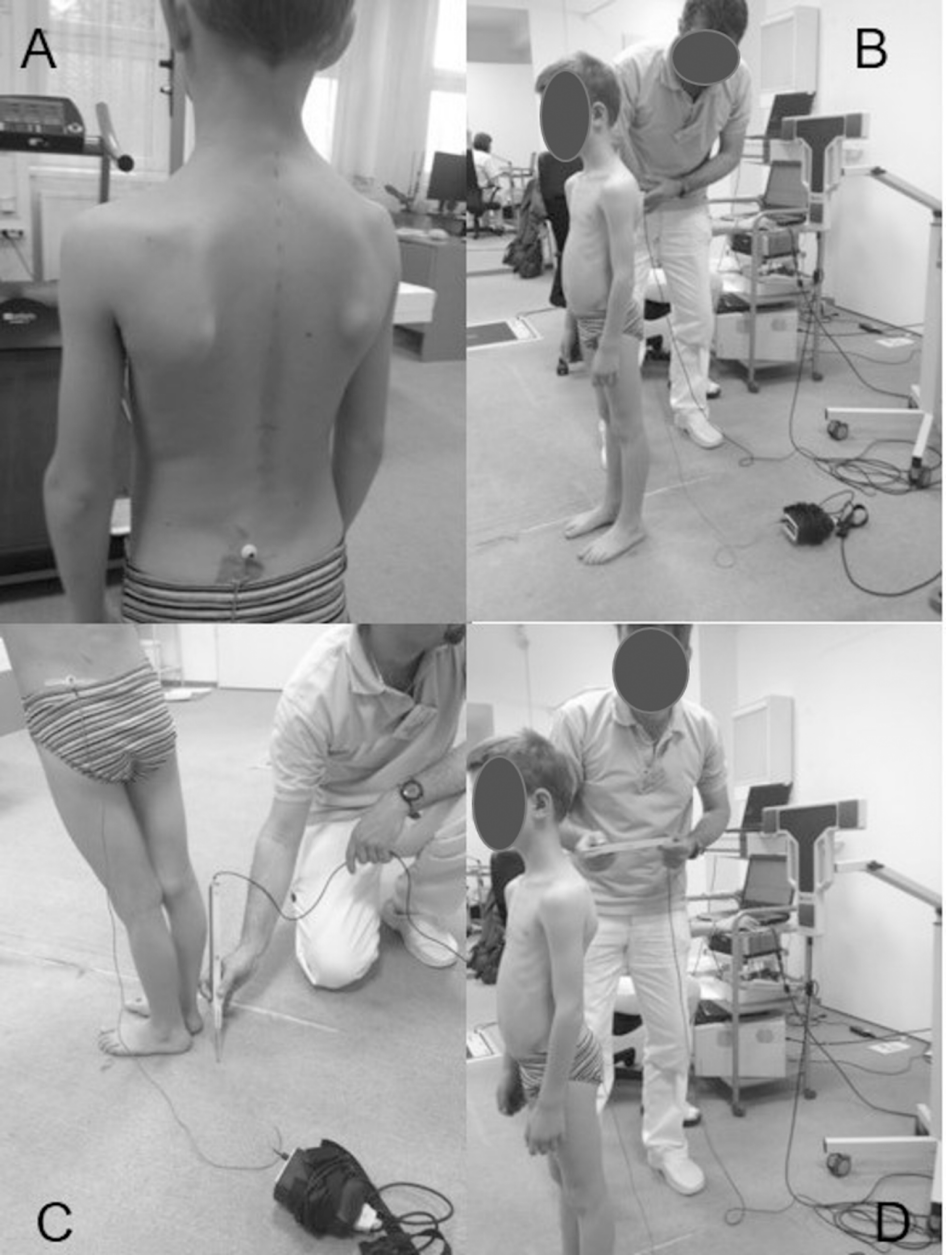
Steps of the measurement. A: placing the reference marker on the skin of the easily palpable part of the pelvis and marking the acromion, the angulus inferior scapulae, the spina iliaca posterior superior, the thoracic 12 (Th12) and the lumbar 1 (L1) on both sides. B: positioning the subject in front of the measurement head with the back facing it. C: calibration: defining the global coordinate system by marking four points on the ground with the pointer stick. D: determining the positions of the spinous processes with the pointer stick between vertebrae C7-S1.

The transmitters of the head emit ultrasound signals in specified intervals (the measuring frequency is 100 Hz), which travel through the air until the receivers record them. The WinSpine program (ZEBRIS Medizintechnik GmbH, Isny, Germany) records and stores the spatial positions of the receivers numerically. The steps of the examination are shown in Fig 2.

Before the first measurement, subjects were asked to take the standing position with lowered arms (Fig 1A). After the first measurement, the subjects could move freely for some minutes, then they were asked to position themselves in the standing position with raised arms (Fig 1B). The spinous processes were determined on each subject and each measurement was performed by the same physiotherapist with experience in palpation to keep inaccuracies at a minimum.

### Measured and calculated parameters

Biplanar X-ray images were processed using the software ImageJ (version 1.54). Cobb angles such as thoracic kyphosis (*RTG_TK*), lumbar lordosis (*RTG_LL*) in the sagittal plane, thoracic curvature (*RTG_TSC*) and thoracolumbar/lumbar curvatures (*RTG_LSC*) in the frontal plane were determined as described in the literature [56]. The test-retest reliability of the determination of Cobb angles with software on X-rays had been analysed by several researchers [57–59]. Based on the results of these studies, interobserver reliability was between 0.75–0.98 and intraobserver reliability was between 0.71–0.98, which are both excellent [57–59]. The accuracy of the method is adequate to determine the sagittal and frontal curvatures of the spine and to follow the changes during treatment or progression[57–59].

The positions of 19 processus spinosus (from C7 to S1) were determined by the ZEBRIS spine examination method. A custom MATLAB-based program (version 2016R, MathWorks, Inc, Natich, MA, USA) fitted a curve on the 19 points by the spline method in both the sagittal and frontal planes. In the sagittal plane, the degree of thoracic kyphosis (*ZEBRIS_TK*) is the angle between the tangential lines over the processus spinosus of Th1 and Th12; the degree of lumbar lordosis (*ZEBRIS_LL*) is the angle between the tangential lines over the processus spinosus of Th12 and L5. In the frontal plane, the angle of thoracic and thoracolumbar/lumbar curvatures was defined by the angle of tangential lines over processus spinosus corresponding to the Cobb-angle boundaries (Table 2) as suggested by Schmid et al [38]. The test-retest reliability (*ICC*) of the ZEBRIS spine examination in cases of children with AIS range from 0.793 to 0.958, which is excellent or very good [47].

**Table 2:** Subject spinal curvature parameters determined by the Cobb method on biplanar X-ray images and by ZEBRIS spinal examination.

		sagittal curvature						thoracic frontal curvature					thoracolumbar/lumbar frontal curvature				
		Cobb angle [deg]		ZEBRIS [deg]		Abs. difference [deg]		convex	Cobb angle		ZEBRIS	Abs. diff	convex	Cobb angle		ZEBRIS	Abs.diff
ID	gender	thoracic	lumbar	thoracic	lumbar	thoracic	lumbar		[deg]	bound-aries	[deg]	[deg]		[deg]	bound-aries	[deg]	[deg]
1	f	13.26	48.10	10.25	37.49	3.01	10.61	right	29.07	T3-T10	16.58	12.49	left	25.06	T11-L4	29.47214	4.41
2	f	32.12	55.79	41.92	40.09	9.80	15.70	right	20.80	T3-T12	19.94	0.86	left	3.58	L1-L4	1.360193	2.22
3	f	37.75	50.86	40.35	39.03	2.60	11.83	right	12.98	T3-T12	11.97	1.01	left	15.45	L1-L5	22.55664	7.11
4	f	13.02	45.10	23.19	41.85	10.17	3.25	right	38.68	T5-T11	28.13	10.55	left	20.90	L1-L5	10.42703	10.47
5	f	49.78	45.60	49.88	41.79	0.10	3.81	left	10.26	T3-T12	10.03	0.23	right	28.85	L1-L5	20.03788	8.81
6	f	16.84	34.00	20.26	36.99	3.42	2.99	right	28.19	T6-T10	28.62	0.43	left	12.51	L1-L5	10.51978	1.99
7	m	54.30	48.94	54.67	53.79	0.37	4.85	right	3.77	T5-T11	1.51	2.26					
8	m	41.66	53.50	43.34	63.15	1.68	9.65	right	6.51	T3-T11	5.91	0.60	left	3.10	L1-L5	0.541092	2.56
9	f	29.30	44.17	20.91	47.32	8.39	3.15	right	25.91	T7-T12	16.86	9.05	left	16.70	L1-L5	9.25996	7.44
10	f	12.73	39.13	12.17	41.12	0.56	1.99						right	15.61	T3-L3	10.85662	4.75
11	f	20.30	32.61	25.75	25.22	5.45	7.39						left	18.61	T10-L4	14.70175	3.91
12	f	12.73	39.13	13.46	31.02	0.73	8.11	right	20.72	T6-T10	24.70	3.98	left	4.58	L1-L5	8.819379	4.24
13	f	4.76	37.31	8.34	32.47	3.58	4.84	right	20.62	T5-T12	26.73	6.11	left	13.86	L1-L5	9.886747	3.97
14	f	23.69	26.24	26.43	26.42	2.74	0.18	right	19.34	T5-T10	20.15	0.81	left	4.69	L1-L5	4.589164	0.10
15	f	19.60	43.10	22.92	39.34	3.32	3.76	right	35.62	T7-T12	30.07	5.55	left	40.40	L1-L4	31.26236	9.14
16	f	33.20	31.88	32.25	30.17	0.95	1.71						left	20.80	T6-L3	11.846	8.95
17	f	20.31	54.69	26.36	67.92	6.05	13.23	left	11.36	T3-T12	7.49	3.87	right	31.45	L1-L5	19.9144	11.54
18	f	20.31	54.69	22.76	44.89	2.45	9.80	left	15.46	T6-T12	5.68	9.78	right	9.36	L1-L5	7.557543	1.80
19	f	50.96	32.08	60.58	29.15	9.62	2.93						left	12.75	T6-L3	16.20323	3.45

### Statistical analysis

The power analysis on the sample size was performed using G*Power (v3.1.9.2) free software (Heinrich Heine University, Düsseldorf, Germany) [60]. The power of a test is the probability of rejecting the null-hypothesis (getting a significant result) when the real difference is equal to the minimum effect size. If the power value is greater than 0.50, the sample size is appropriate [60].

The basic statistical features such as the mean, standard deviation (SD) and 95% confidence intervals (CI) were determined both in the angle values determined by the ZEBRIS spine examination method (*ZEBRIS_TK*, *ZEBRIS_LL*, *ZEBRIS_TSC*, *ZEBRIS_LSC)* and in the angle values determined by the Cobb method on X-ray images (*RTG_TK*, *RTG_LL*, *RTG_TSC*, *RTG_LSC*). Statistical analysis was performed using IBM SPSS software (ver. 24, IBM corporation) with the level of significance set at *α* = 0.05.

The systematic method of comparing the sagittal and frontal spinal curves determined by the non-invasive spine measurement method and by the Cobb method on X-ray images is considered the gold standard. Schmid et al. [38] performed a comparison with linear regression only. In the present study, the validity of the ZEBRIS spine examination method was analysed with Bland-Altman analyses between the sagittal and frontal spinal curvature parameters calculated from data determined by the ZEBRIS spine examination method (*ZEBRIS_TK*, *ZEBRIS_LL*, *ZEBRIS_TSC*, *ZEBRIS_LSC)* and data obtained by the Cobb method on X-ray images (*RTG_TK*, *RTG_LL*, *RTG_TSC*, *RTG_LSC*) [61]. We used the complete Bland-Altman method: in addition to the parameters of linear regression (Pearson r-value squared, slope of regression line, intercept), we calculated the parameters of the Bland-Altman method such as the mean, limit of agreement and 95% CI of bias and plotted the Bland-Altmann diagram with the following parameters: sum of squared error, reproducibility coefficient and values of Kolmogorov-Smirnov test [61].

In the present study, it was not possible to examine the effect of soft tissue thickness with the radio-opaque method and to measure the soft tissue thickness with a calliper. BMI index can be calculated from the available anthropometric data (Table 1), from which index BMI% can also be calculated using the percentile table. The percentile table shows what percent (BMI%) of children of the same gender and age have a lower BMI than the measured subject. The 50th percentile is the average body mass index [48,49]. According to the literature [48,49], there is a linear relationship between the percentile table value and the soft tissue thickness, thus, for modelling the effect of soft tissue thickness, the Pearson correlation between the BMI percentile (BMI%) [48,49] and the absolute difference between spinal curvature angles determined by the two measurement methods was calculated. Validity coefficients were defined as follows: 0.81≤*r*≤1 excellent, 0.61≤*r*≤0.80 very good, 0.41≤*r*≤0.60 good, 0.21≤*r*≤0.40 fair, 0.00≤*r*≤0.20 poor [61].

## Results

The power of the sample size is 0.79, indicating that the sample size is large enough to detect significant differences between the two methods [60].

The anthropometric data (Table 1), the frontal and sagittal plane angle values calculated with the two methods (*RTG_TK*, *RTG_LL*, *RTG_TSC*, *RTG_LSC*, *ZEBRIS_TK*, *ZEBRIS_LL*, *ZEBRIS_TSC*, *ZEBRIS_LSC*) and the differences between these two sets of values (Table 2) are given for each subject. The means, standard deviation and 95% CI values of *TK*, *LL*, *TSC* and *LSC* as determined by the two methods were also given in (Table 3). Significant bias (difference) is shown for *TK* (-2.6°, *p* = 0.02) and for *LSC* (3.2°, *p* = 0.02); however, bias for *LL* (2.5°, *p* = 0.16) and *TSC* (3.0°, *p* = 0.05) is not significant (Table 4). The limit of agreement for differences calculated by the standard deviation of differences is much wider (greater than 17°) than the 95% confidence interval calculated from the standard error of differences (less than 7.2°) (Table 4).

**Table 3:** Means, standard deviations and 95% CIs of compared parameters.

	mean	standard deviation	95% CI	
			lower bound	upper bound
*RTG_TK*	26.66	14.58	19.64	33.69
*RTG_LL*	43.00	9.00	38.66	47.33
*RTG_TSC*	19.95	10.23	15.02	24.88
*RTG_LSC*	16.57	10.23	11.64	21.50
*ZEBRIS_TK_*	29.25	15.24	21.91	36.60
*ZEBRIS_LL*	40.49	11.45	34.97	46.00
*ZEBRIS_TSC*	16.96	9.48	12.39	21.53
*ZEBRIS_LSC*	13.32	8.59	9.18	17.47

**Table 4:** Results of Bland-Altman analysis on spinal curvature angles.

	Regression line			Bias		
	*r*^*2*^	slope of regression line	intercept (°)	mean (°)	limit of agreement (°)	95% confidence interval (°)
*TK*	0.91	1.000	2.64	2.6	]11.0; -6.3[	]4.8; -0.4[
*LL*	0.58	0.970	-1.27	-2.5	]12.0; -17.0[	]1.1; -6.1[
*TSC*	0.72	0.790	1.26	-3.0	]7.7; -14.0[	]0.0; -6.0[
*LSC*	0.71	0,710	1.62	-3.2	]7.6; -14.0[	]0.5;- 6.0[

Limit of agreement equals the range of the bias ± 1.96 times the standard deviation of the differences; 95% confidence interval equals the range of the bias ± 2.1 times standard error of differences. For these calculations, we have 18 degrees of freedom and t = 2.1.

**Fig 3:**
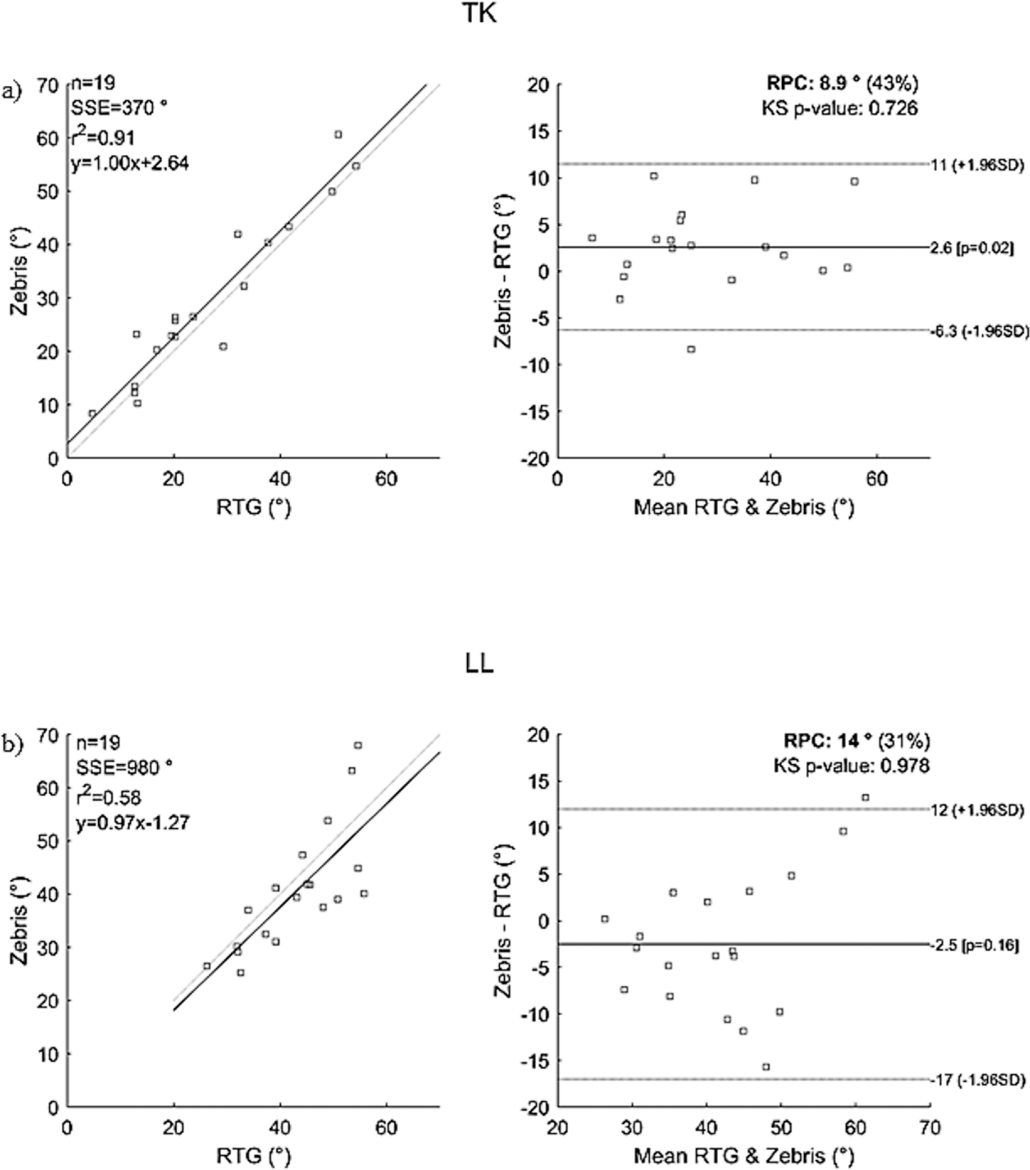
Bland-Altman plots of spinal curvature angles in the sagittal plane comparing the results of the two measurement systems. a: sagittal thoracic spinal curvature angle (*TK*), b: sagittal lumbar spinal curvature angle (*LL*). Comments: *SSE*—sum of squared error; *r*^*2*^—Pearson r-value squared; *RPC* (%)—reproducibility coefficient and % of values; *ks*—Kolmogorov-Smirnov test for normality of differences, all values are higher than 0.05 and the distribution is Gaussian.

**Fig 4:**
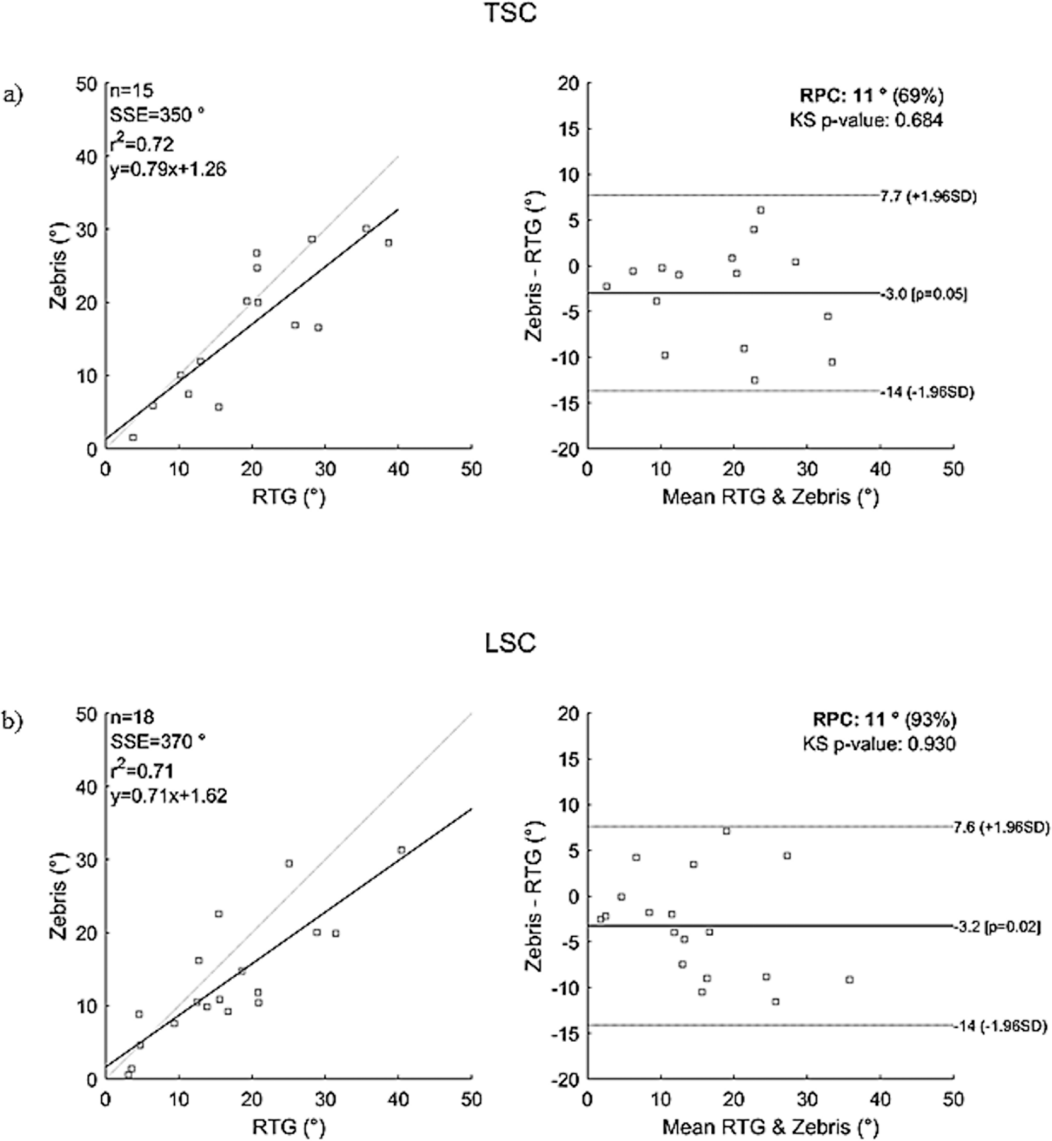
Bland-Altman plots of spinal curvature angles in the frontal plane comparing the results of the two measurement systems. a: frontal thoracic spinal curvature angle (TSC), b: frontal thoracolumbar/lumbar spinal curvature angle. Comments: *SSE*—sum of squared error; *r*^*2*^—Pearson r-value squared; *RPC* (%)—reproducibility coefficient and % of values; *ks*—Kolmogorov-Smirnov test for normality of differences, all values are higher than 0.05 and the distribution is Gaussian.

The results of Bland Altman analysis are plotted separately for each of the spinal curvature angles in the sagittal (*TK*, *LL*) (Fig 3) and frontal planes (*TSC*, *LSC*) (Fig 4). The linear regression for *TK* draws the line of equality, with a slope of 1.00 and the intercepts are below 5 degrees (Table 4). The correlation between the *TK* values determined by the two methods is significant (p = 0.000) and excellent (*r*_*TK*_ = 0.95) (Fig 3A). However, the intercept value of the fitted line for the parameter *LL* is significantly nonzero (1.27°, *p* = 0.002), the slope is below one (0.97, *p* = 0.000) (Table 4) and the correlation is very good (*r*_*LL*_ = 0.76) (Fig 3B). The linear regression for *TSC* and *LSC* shows that the slopes are below one (0.79 and 0.71, respectively) and the intercept values are below 5 degrees (Table 4). The correlation between the *TSC* and *LSC* values determined by the two methods is significant (*p* = 0.000) and excellent (*r*_*TSC*_ = 0.85, *r*_*LSC*_ = 0.84) (Fig 4). One data point (5.2%) at high *LL* values (*RTG_LL* = 54.69°, *ZEBRIS_LL* = 67.92°) is out of the limit of agreement (12.0; -17.0) (Fig 4B). All the data for *TK*, *TSC* and *LSC* are within the limit of agreement. (Figs 3A and 4). Reproducibility, which refers to the variation in measurements made on a subject due to different measuring methods [61], is under 14° (Figs 3 and 4).

Pearson correlation coefficients between the percentile body mass index of children [48][49] (Table 1) and of the absolute difference between the sagittal thoracic and both frontal spine curvatures determined by the two measurement methods (Table 2) are below 0.25 (*r*_*TK*_ = -0.07 *p*_*TK*_ = 0.02; *r*_*TSC*_ = 0.25 *p*_*TSC*_ = 0.01, *r*_*LSC*_ = 0.16, *p*_*LSC*_ = 0.03), which means the correlation is poor to fair. However, in the case of the sagittal lumbar angle the correlation is good (*r*_*LL*_ = 0.56, *p*_*LL*_ = 0.04).

## Discussion

Using biplanar radiographical and ZEBRIS spine examination, the aim of this study is to evaluate the static validity of the ZEBRIS spine examination method in the determination of spinal curvatures in the sagittal and frontal planes in patients with AIS. Sagittal and frontal angles could be estimated with reasonable accuracy by the ZEBRIS spine examination, whereas thoracolumbar/lumbar curvature angles were systematically underestimated. The present study has the novelty of the validation of sagittal and frontal angle values by the ZEBRIS spine examination method with angle values determined by the gold standard Cobb method on X-ray images. The validity was analysed by Bland-Altman analyses [61].

There are only a few studies that have evaluated the marker-based motion analysis system for the estimation of spinal curvatures in the sagittal plane using radiography in healthy subjects and these used regression analysis only [62–65]. There is only one study that evaluated the marker-based motion analysis system for the estimation of spinal curvatures in the sagittal and frontal planes [38]; however, they also used regression analysis only. A comparison of this previous [38] and our present study is summarized in Table 5. In the previous study [38], the positions of the processus spinosus of 11 vertebrae are determined from reflective markers attached to the skin over the processus spinosus by a VICON motion analysis system. In the present study, the positions of the processus spinosus of 19 vertebrae are determined from the position of a pointer stick pointed at the skin over the processus spinosus by the ZEBRIS ultrasound-based motion analysis system.

**Table 5:** Comparison of subjects, methods, data and spinal curvature angles of present and previous research.

subjects			**Previous research** [38]	**Present research**
	number		10	19
	gender		8 females and 2 males	17 females and 2 males
	age [years]		14.8±1.3 (12–16)	14.5±2.1 (8–16)
	body mass [kg]		55.3±12.7 (38.5–85.5)	50.5±10.6 (30–67)
	body height [cm]		165±10 (152–184)	165.4±11.1 (140–182)
	Lenke type [6]		1 and 3	1 and 3
Motion Analyses System (MAS)	type		VICON with 12 cameras	ZEBRIS CMS-HS
	markers		reflective markers with diameters 9–14 mm	sticker with diameters 4 mm
	investigated processus spinosus		C7, T3, T5,T7, T9, T11, L1-L5	C7-S1
Position			standing	standing
Cobb angles [°]	sagittal thoracic		17.5±10.1	26.7±14.6
	sagittal lumbar		47.3±16.8	43.0±9.0
	frontal thoracic		44.4±17.7	19.9±10.2
	frontal thoracolumbar/lumbar		36.9±12.6	16.6±10.2
angles determined by motion analysis system (MAS) [°]	sagittal thoracic		no numerical data	29.3±15.2
	sagittal lumbar		no numerical data	40.5±11.5
	frontal thoracic		no numerical data	17.0±9.5
	frontal thoracolumbar/lumbar		no numerical data	13.3±8.6
Comparison			regression analysis	Bland-Altman method
results of regression analysis RTG = x*MAS+y	sagittal thoracic	x	0.897	1.00
		y	-9.06	2.64
		R^2^	0.901	0.91
	sagittal lumbar	x	0.946	0.970
		y	-2.45	-1.27
		R^2^	0.681	0.58
	frontal thoracic	x	0.764	0.790
		y	-26.300	1.26
		R^2^	0.700	0.72
	frontal thoracolumbar/ lumbar	x	0.863	0.710
		y	-17.6	1.62
		R^2^	0.521	0.707
mean of bias [°]	sagittal thoracic		not calculated	2.6
	sagittal lumbar		not calculated	-2.5
	frontal thoracic		not calculated	-3.0
	frontal thoracolumbar/lumbar		not calculated	-3.2
limit of agreement [°]	sagittal thoracic		not calculated	]11.0; -6.3[
	sagittal lumbar		not calculated	]12.0; -17.0[
	frontal thoracic		not calculated	]7.7; -14.0[
	frontal thoracolumbar/lumbar		not calculated	]7.6; -14.0[

Based on the Bland-Altman analysis, it can also be stated that the measurement results have a normal distribution.

Let’s analyse the sagittal spinal curvature (*TK* and *LL*) first. The results of the regression analysis of both studies are similar (Table 5), which supports our main findings: the correlations between the sagittal curvature angles range (*r*_*TK*_ = 0.95; *r*_*LL*_ = 0.76;) are excellent and very good. (Table 4). The correlation is similar to those of previous studies in healthy subjects [62–65] as well. Our results supported the results of a previous study [38]: sagittal lumbar spinal curvatures derived from the processus spinosus compared to angles derived by the Cobb-method are underestimated. This is confirmed by the negative sign of the bias (-2.5° with 95% confidence interval, range 1.1° to -6.1°) (Table 4); the value determined with the Cobb method is greater than the angles derived from the processus spinosus. The results of Bland-Altman analysis (Fig 3) showed that the accuracy of the estimation of sagittal lumbar curvature angles was worse (95% confidence interval and limit of agreement are wider) than that of thoracic curvature angles. This is shown clearly by the fact that the *RPC* value of angle *LL* is below 14° (Fig 3B). These findings are in agreement with the results of the validation of the Spinal Mouse [36] and of the skin marker based method [38]. The Bland-Altman diagram of *LL* (Fig 3B) shows that the differences at over 50 degrees of sagittal lumbar curvature angle are increased, which is in agreement with the results of previous studies [36,38]. Previous studies [43,44] have analysed the test-retest reliability of the ZEBRIS spine examination method and found that the level of reliability of the *TK* angle is higher than that of the *LL* angle and that there is very good reliability in thoracic angles and good reliability in lumbar angles. The analysis of test-retest reliability of the ZEBRIS spine examination method in children with AIS shows that interobserver and intraobserver reliabilities of lumbar lordosis are very good, and interobserver and intraobserver reliabilities of thoracic kyphosis are excellent [47]. The smallest correlation (*r* = 0.76) was found in *LL* angles determined with the two examination methods. Previous studies [62–65] found that the reliability of *TK* is better than the reliability of *LL*. Schmid et al. [38] clearly regarded the thicker soft tissue present in the lumbar region as the reason for this, based on comparisons made with measurements using radio-opaque markers. According to data found in the literature [38–41,43,44,62–65] the paravertebral muscles in the lumbar section are always more emerged than in the thoracic section. Lumbar fat tissue could be a reason why the determination of the positions of the deeper bony formulations are more inaccurate. Our measurements can support this hypothesis only indirectly: the correlation between the percent of body mass index and the difference between the *LL* values determined with the two methods is only good (*r* = 0.56). This indicates that the palpation of the processus spinosus of the lumbar region requires more attention in children with a higher body mass index.

Let’s now analyse the frontal spinal curvature (*TSC* and *LSC*). The present study shows that the correlation of the frontal thoracolumbar/lumbar curvature angles is excellent (*r*_*LSC*_ = 0.85) (Table 4). The bias of the frontal thoracolumbar/lumbar curvature angles was -3.2° with a 95% confidence interval of 0.5° to -6.0° (Table 4), which is smaller than the error determined with computerized photogrammetry by Aroeira et al. [37] (5.1°). The frontal thoracolumbar/lumbar curvature angles derived from the processus spinosus tends to give lower values compared to the values as determined by the Cobb method (Fig 4A), which is indicated by the negative sign of the bias (Table 4). The reasons for such clear underestimation can include some deformations developing in the vertebrae over time [66] and the rotational deformities in AIS patients (i.e., axial rotation and intrinsic axial torsional deformity of the vertebrae)[38]. The correlation of the frontal thoracic curvature angles was excellent (*r*_*LSC*_ = 0.85) (Table 4). The bias was 3.0° with a 95% confidence interval of 0.0° to -6.0°, which agrees with the error (2.9) determined with computerized photogrammetry by Aroeira et al [37]. The frontal thoracic curvature angles derived from the processus spinosus tend to give lower values compared to the angles determined by the Cobb method (Fig 4A). Similar to the lumbar section, this is due to the deformations developing in the vertebrae [66] and to the rotational deformities in AIS patients [38].

One advantage compared to the computerized photogrammetry method using a cheap digital camera by Aroeira et al [37], is that it can measure sagittal angles along with the frontal curvature angles with a single measurement. The bias of the frontal thoracolumbar/lumbar curvature angles (3.2°) is significantly lower than in the case of a computerized photogrammetry method (5.1°). Aroeira et al [37] also found that the main drawback of the photographic method is that the average duration of the measurement (positioning, surface marking, photographic exposure and one curve measurement) is 28 minutes, while the average duration of the ZEBRIS spine examination (positioning, palpation of the anatomical points and processus spinosus and one curve measurement) is 17 minutes. The repetition accuracy of the method described in the article by Aroeira et al [37] is unknown and only the differences between the angular values determined with the two methods are known, while other factors affecting accuracy are not.

Based on the Bland-Altman analysis results, the utility of the ZEBRIS spine examination method for a comprehensive evaluation of treatment effects as a non-invasive method could be recommended. The adequacy of the method for the follow-up of patients with AIS is confirmed not only by the small deviation (≤3.5°) from the sagittal and frontal curvatures determined with the gold standard method on X-rays but also by the high or excellent test-retest reliability (ICC≥0.793) determined on AIS patients [47]. During the examination of scoliosis, attention has to be paid to the distortion of the sagittal plane curvatures, which can be manifested primarily in the flattening of the thoracic kyphosis [7] and could be a sign of progression [8]. The flattening of the thoracic kyphosis in the sagittal plane as the secondary sign of progression could be suitable for accurately assessing the progression of scoliosis because of the excellent test-retest reliability [47] and low bias (2.6°), as well as the excellent correlation (*r* = 0.95), which ensures the accuracy of the *TK* values.

The present study has some limitations: the validation process was performed on the same day; however, measurements were not performed simultaneously; the pointing accuracy for processus spinosus by the pointer was not investigated; the underestimation of spinal curvature in the frontal plane suggests that it might be possible to perform a systematic correction of the angle values determined by the ZEBRIS spine measurement method on a larger sample size in the future.

## Conclusions

This study fills a gap in the literature because it validates the ZEBRIS spine examination method with the sagittal and frontal spinal curvature angles determined by the gold standard Cobb method on X-ray images in patients with AIS. The thoracic and lumbar spinal curvature angles in the sagittal plane were measured with reasonable accuracy. The thoracolumbar/lumbar spinal curvature angles in the frontal plane were systematically underestimated, mainly due to the rotational and pathological deformities of the scoliotic vertebrae.

ZEBRIS spinal examination cannot replace the biplanar X-ray examination for the visualization of spinal curvatures in the sagittal and frontal planes and the rotation of vertebral bodies during the diagnosis and annual evaluation of progression. Practice recommendations state that taking X-ray images is allowed only once per year [20]. However, the ZEBRIS spine examination method could be used for follow-ups several times a year, e.g., examining the effectiveness of various therapies, thus reducing the radiation exposure to patients. Between the two biplanar radiological examinations, the numerical results provided by ZEBRIS non-invasive spinal examination equipment can provide an objective view of the spine curvature during standing and indirectly about the effectiveness of therapy.
